# Agricultural burned area detection using an integrated approach utilizing multi spectral instrument based fire and vegetation indices from Sentinel-2 satellite

**DOI:** 10.1016/j.mex.2022.101741

**Published:** 2022-05-29

**Authors:** Monish Vijay Deshpande, Dhanyalekshmi Pillai, Meha Jain

**Affiliations:** a Indian Institute of Science Education and Research Bhopal (IISERB), Bhopal, India; b Max Planck Partner Group (IISERB), Max Planck Society, Munich, Germany; c School for Environment and Sustainability, University of Michigan, Ann Arbor USA

**Keywords:** Agricultural burned area mapping, Sentinel-2, Planet data, Google Earth engine

## Abstract

This study presents a methodology that focuses on detecting agricultural burned areas using Sentinel-2 multispectral data at 10 m. We developed a simple, locally adapted, straightforward approach of multi-index threshold to extract post-winter agricultural burned areas at high resolution for 2019-21. Further, we design a new method for virtual sample collection using already validated fire location data and visual interpretation conditioned using strict selection criteria to improve sample accuracy. Sampling accuracy showed near-perfect agreement with an average Cohen's Kappa value of 0.98. We retrieved monthly ABAs at a resolution of 10 m, and these products were validated against reference burned sample plots identified using visual interpretation of Planet (3m) satellite data. Overall, we found that our method performed well, with an F1 score of 83.63% and low commission (20%) and omission (7%) errors. When compared to global burnt area products, validation accuracy demonstrated an exceptional subpixel scale detecting capability. The study also addresses the complexity of residue burnings and burn signatures’ volatile nature by performing multilevel masking and temporal corrections.

• A novel remotely sensed data aided virtual sampling approach to acquire burned and unburned samples.
• An integrated method to extract smallholder agricultural burned area using Sentinel-2 multispectral data at a high resolution of 10 m

## Graphical abstract

**Figure:**
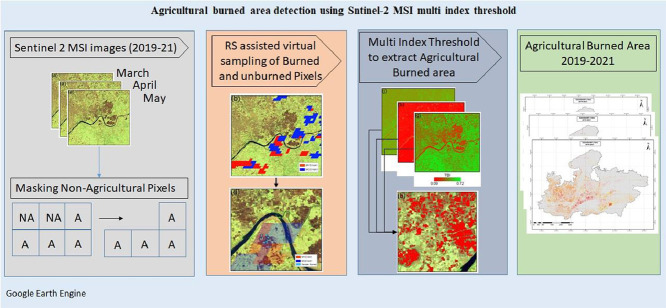

Specifications table

**Table:** 

Subject Area;	Environmental Science
More specific subject area;	Remote Sensing of Environment
Method name;	Agricultural burned area detection using Sentinel-2 MSI multi index thresholding
Name and reference of original method;	Roteta, E., Bastarrika, A., Padilla, M., Storm, T., & Chuvieco, E. (2019). Development of a Sentinel-2 burned area algorithm: Generation of a small fire database for sub-Saharan Africa. Remote Sensing of Environment, 222 (November 2018), 117. https://doi.org/10.1016/j.rse.2018.12.011
Resource availability;	Sentinel-2 MSI: MultiSpectral Instrument, Level-2A
	Global PALSAR-2/PALSAR Forest/Non-Forest Map
	Esri 2020 Land Cover Downloader
	MOD14A1.006: Terra Thermal Anomalies & Fire Daily Global 1km
	MYD14A1.006: Aqua Thermal Anomalies & Fire Daily Global 1km
	FireCCI51: MODIS Fire_cci Burned Area Pixel product, version 5.1
	MCD64A1.006 MODIS Burned Area Monthly Global 500m
	Satellite Imagery and Archive | Planet

## Background

We present a methodology adapted for Sentinel -2 MSI derived burn indices to detect agricultural burned area (ABA) using the Google Earth Engine (GEE) platform. The existing global data products significantly underestimate the agricultural burned area due to the diverse nature of agricultural fields and the spread of fire activities [1,^21^,29]. Small agricultural fires, which are frequent across India's smallholder terrain, have been proven to be missed by coarse resolution sensors, including MEdium Resolution Imaging Spectrometer (MERIS), Moderate Resolution Imaging Spectroradiometer (MODIS), and Advanced Very High-Resolution Radiometer (AVHRR). Considering spatial and temporal resolutions of smallholder burning, Sentinel-2 with high resolution (10 m) can provide an optimal solution for the detection of ABA.

Burned scar sampling, an essential factor for algorithm training and validation, is popularly done by selecting ground control points or visual interpretation, but both have limitations [13,^14^,^22^,25]. To overcome the limitations of area coverage and visual prediction errors from ground control points and visual identification, respectively, we propose a novel approach of virtual sampling. Our study explored the potential of the Terra Thermal Anomalies & Fire Daily Global 1 km (MOD14A1.006) and Aqua Thermal Anomalies & Fire Daily Global 1 km (MYD14A1.006) datasets combined with visual interpretation to overcome the field sampling limitations. The method used the standard MODIS Fire and Thermal Anomalies product to support the visual identification of burn scars for sampling. The technique can help significantly to reduce the dependency on ground sampling. The approach generates high-resolution regional agriculture burnt area (ABA) products in GEE using Sentinel -2 MSI derived indicators and well-conditioned virtual sampling. These products can be utilized to address the uncertainties in the estimates of stubble burning emissions from mostly unexplored areas across Central India.

Below we describe the data and methods used in this paper for sampling training and validation data (Section 1), mapping burned area using Sentinel-2 imagery (Section 2), and validating our burned area product (Section 3). Table 1 lists the datasets used and for which specific purpose.

**Table 1:** An Overview of datasets used in this study.

No.	Dataset	Usage	Source
1.	Sentinel-2 MSI: MultiSpectral Instrument, Level-2A	Burned Scar Identification, Quantification, Validation	Sentinel-2 MSI: MultiSpectral Instrument, Level-2A
2.	Global PALSAR-2/PALSAR Forest/Non-Forest Map	Forest and Non-forest Mask	Global PALSAR-2/PALSAR Forest/Non-Forest Map
3.	ESRI LULC 2020	Mask	Esri 2020 Land Cover Downloader
3.	MOD14A1.006: Terra Thermal Anomalies & Fire Daily Global 1 km	Burned Scar Identification, Validation	MOD14A1.006: Terra Thermal Anomalies & Fire Daily Global 1km
4.	MYD14A1.006: Aqua Thermal Anomalies & Fire Daily Global 1 km	Burned Scar Identification, Validation,	MYD14A1.006: Aqua Thermal Anomalies & Fire Daily Global 1km
5.	FireCCI51: MODIS Fire_cci Burned Area Pixel product, version 5.1	ABA Comparison, Validation	FireCCI51: MODIS Fire_cci Burned Area Pixel product, version 5.1
6.	MCD64A1.006 MODIS Burned Area Monthly Global 500 m	ABA Comparison, Validation, Emission estimates and comparison	MCD64A1.006 MODIS Burned Area Monthly Global 500m
7.	PlanetScope 4-band multispectral basic and orthorectified scenes	ABA Validation	Satellite Imagery and Archive | Planet

## Collection of training and validation data

**Fig. 1:**
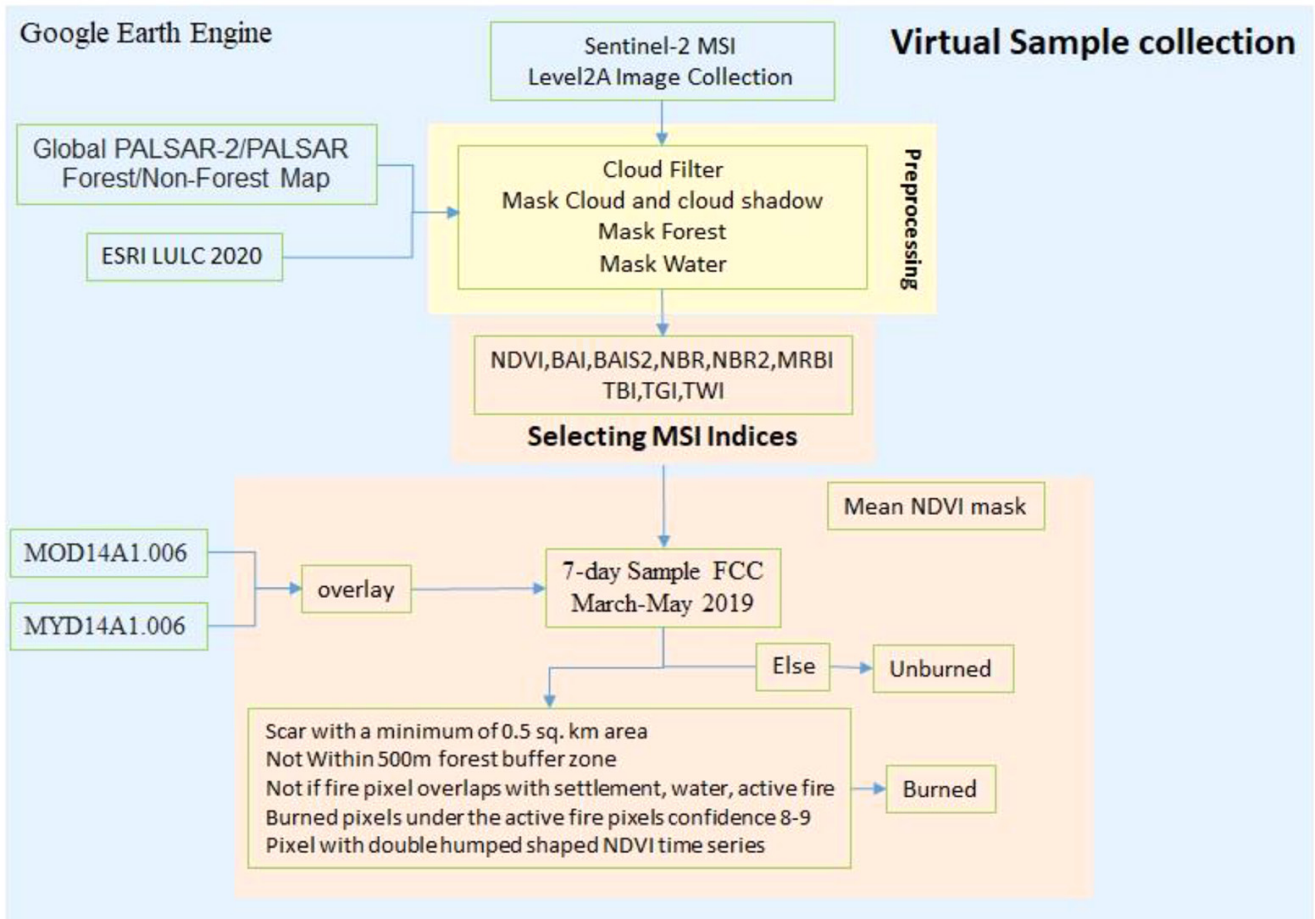
Remotely sensed data assisted virtual sampling of agriculture burned and unburned pixels.

To collect ground truth data on burned and unburned pixels, we developed a novel virtual sampling methodology (Fig. 1) that uses active fire data sets to target likely regions with burning for visual interpretation of Sentinel-2 imagery. The following sub-sections provide details of the datasets used and the sampling method that we developed for this study.

### Remotely sensed data assisted virtual sampling of agriculture burned and unburned pixels

For the virtual sampling method, we used satellite products from MODIS 4 and 11-micrometre radiances to create daily fire mask composites at 1 km resolution: MYD14A1.006 (Aqua Thermal Anomalies & Fire) and MOD14A1.006 (Terra Thermal Anomalies & Fire). These databases have been previously used to capture forest fires and agricultural residue burning [7,^27^,31]. Daily fire mask composites for 2019 from March to May were used to perform assisted sampling of S2A burnt pixels. The time period is chosen following the winter crop harvest and associated burning of residues in our study region.

Both fire products are ideal for fire monitoring considering the short temporal resolution of fires and the temporal coverage (day and night) of MODIS products. Based on the Sentinel-2 repeat cycle and study area coverage, we generated seven-day composites of S2A datasets for March, April and May 2019 (more details about S2A imagery and pre-processing in Section 2.1). Similarly, seven-day composites were created from daily MODIS fire products for detecting burned scars. A false colour composite (FCC) of S2A data was created with short infrared, near-infrared and green bands (R: SWIR2, G: NIR, B: GRE) for better visual interpretation of burned areas. After analysing visualisation parameters in GEE, maroon coloured burned patches were analysed for further sampling using active fire products [11,13]. The S2A seven-day composite was overlaid with seven-day composites of MOD14A1.006 and MYD14A1.006 to match observed fire activities from the global active fire products and visually detected burned scars from S2A data.

**Fig. 2:**
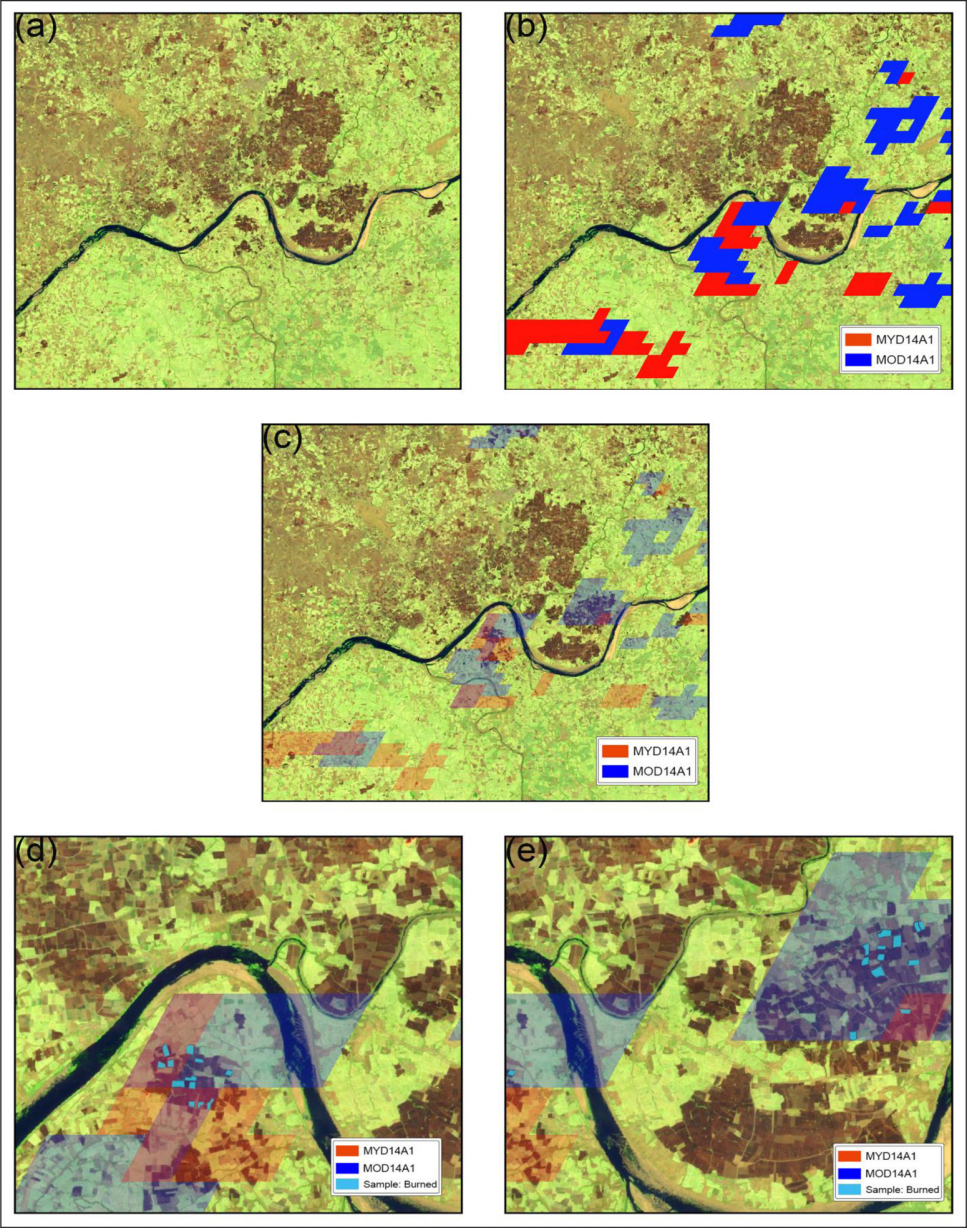
Remotely sensed data assisted virtual sampling strategy for burned areas. (a) FCC image (R:B12,G:B8,B:B3) showing burned scars.(b)FCC overlaid with MYD14A1 and MOD14A1 fire location data. (c) Suitable sampling sites under MYD14A1 and MOD14A1 tiles. (d) and (e) Sample plots selection based on sampling criteria.

Fig. 2 gives an overview of the sampling strategy for detecting burned areas. We formulated criteria to identify agricultural burned areas using visual interpretation. Burned scars from S2A seven-day composites overlapped with seven-day composites of MODIS fire products were considered for sampling if all of the sampling criteria (Fig. 1) were met. Since MODIS has a ∼50% probability of detecting a fire with an area of at least 18 ha [30], only burn scars with a minimum of 0.5 km^2^ (∼50 ha) area were considered for sampling. We have not considered burn scars within 500 m of forested area to avoid the impact of forest fires on our products. Additionally, active fire pixels that overlapped with settlements bigger than 1 km^2^, isolated power plants, factories with chimneys, mining structures, and unmasked water bodies were excluded to avoid impacts from non-agricultural fires and water pixels. Pixels with active flame or covered by smoke were also excluded to eliminate the effect of smoke and fire on associated spectral indices. MOD14A1.006 and MYD14A1.006 fire products provide nine classes, with classes 7-9 denoting fire pixels based on low to high fire confidence, respectively. We only considered fire pixels with high confidence (classes 8 and 9) to detect burned pixels of S2A images. To ensure that the sampled burned pixels belonged to agricultural land, we restricted our sampling to pixels that showed a double hump-shaped phenology in the Normalized Difference Vegetation Index (NDVI) time series across two cultivation seasons [28]. We calculated the NDVI time series for all study years using S2A data.

Sample polygons were created to make a feature collection in GEE for further analysis. Due to GEE limitations, approximately 100 such polygons with burned samples were collected per composite for 2019. Due to insufficient active fire pixels during the first half of March 2019, we did not consider the first two S2A composites for sampling. We collected 1160 burned sample polygons over the sampling period (March-May 2019), with approximately 40 burned pixels for each polygon.

Harvested or ploughed croplands tend to show comparable spectral characteristics to burned areas [13,22]. To address this, unburned open field samples in proximity to the burned samples were collected by visual interpretation of S2A images. In addition, unburned samples from unharvested croplands, vegetation, settlements, bare riverbanks and unmasked water bodies were also collected. Other non-burnable features, including topographical shadows, rocky surfaces and transportation routes (roads, railways) were also considered in the unburned sample. Approximately 40 polygons from each subclass were extracted to ensure their proper representation in the sample dataset. We maintained the pixel number in each polygon to be the same as that of the burned samples (approximately 40 pixels/polygon), and 3246 unburned sample polygons were created over the sampling period.

### Accuracy assessment of sampling strategy

We performed image classification on all S2A seven-day image composites using the burned and unburned samples that we collected and assessed the accuracy of the sampling strategy. The smileRandomForest algorithm developed by GEE was applied to train a decision forest classifier. The unburned samples were further divided into harvested/open field, vegetation and non-burnable classes to increase the classification accuracy and to identify the pixel classes that are most likely to get misclassified as burned. In GEE, we performed hyperparameter tuning with each sample set to select the optimal number of decision trees and limit the number to 200. A 60% threshold was applied to randomly choose sample polygons for training and validation of the classifier. All the three subclasses of unburned samples were processed as a single unburned class for further analysis.

## Detecting agricultural burned signatures using satellite imagery

### Satellite dataset and pre-processing

We used the Sentinel-2, Level 2A (S2A) product offered by the European Space Agency (ESA) for ABA detection. The data were freely available on the GEE platform. Level 2A data were surface reflectance, bottom of the atmosphere corrected products computed from associated Level-1C data using the algorithm Sen2Cor [16]. The data were provided as 100 × 100 km^2^ tiles with UTM/WGS84 projection [18]. The spatial resolution difference between bands (10 m for spectral bands 'B2-B4′ and 'B8 and 20 m for spectral bands ‘B6’, ‘B7’, ‘B8A’, ‘B11’, ‘B12’), was resolved by the GEE's scaling algorithm that resamples spatial resolution during data export [5], and we exported all bands and indices at 10 m resolution. The S2A images were filtered for those that had less than 10% of cloud cover, and we used the 'QA60′ band at 60 m spatial resolution to mask the remaining cloud and cloud shadows. Removing cloud shadows is particularly important given that they have a similar spectral signature as burn scars across fire indices. We additionally visualized all images and excluded those images with haze-covering burned scars. In total, 3,522 S2A tiles were processed to produce our final dataset.

We masked land covers that are not associated with agriculture but can affect burned area detection using several datasets. To mask forests, water and flooded vegetation, we used the ESRI 2020 Global Land Use Land Cover product (10 m) [8]. A subset for Madhya Pradesh (MP LULC 2020) was extracted from the global dataset to create a mask for water, tree and flooded vegetation classes. We also applied an additional water mask using the water class from the Global PALSAR-2/PALSAR Forest/Non-Forest Map, provided by the Japan Aerospace Exploration Agency Earth Observation Research Center (JAXA EORC), for 2017. In addition, the Modified Normalized Difference Water Index (MNDWI) threshold was also used to mask water pixels. The water pixel threshold was determined by random sampling of waterbodies from S2A images.

### Spectral indices and image transformation

Due to the diverse nature of agricultural fields and the spread of fire activities during the study period, several indices designed explicitly for burned area detection were analysed along with chlorophyll, moisture and brightness indices (Table 2). Three bands from the visual spectrum (VIS: 'B2, B3 and B4′), a near-infrared band (NIR: 'B8′), three red edge bands (RE: 'B6′, 'B7′ and 'B8A'), a short wave infrared short reflectance band (SWIR1: 'B11′) and a short wave infrared long reflectance band (SWIR2: 'B12′) were used to compute indices needed to detect burned area. Charcoal and ash sensitive indices like Normalised Burn Ratio (NBR), Normalised Burn Ratio 2 (NBR2), Burn Area Index (BAI), Sentinel 2 specific Burn Area Index (BAIS2) and Mid-Infrared Burn Index (MIRBI) were used to identify the burned scars immediately after the fire event [4,10,15,17,24,26]. NDVI was also used to exclude false-positive signatures from ash settlement on unburned fields [19]. We used Tasseled Cap Transformation (TCT) derived Brightness, Greenness, and Wetness indices to integrate a more extensive range of spectral information [23]. After calculating the spectral indices, we extracted their values for all 4406 sample polygons for further analysis.

**Table 2:** List of spectral indices and image transformation techniques used in this study.

Index Full Name	Abbreviation	Equation	Reference
Normalized Difference Vegetation Index	NDVI	$\frac{\rho_{NIR\;}-\rho_{RED\;}}{\rho_{NIR\;}+\rho_{RED\;}}$	[24]
Burn Area Index	BAI	$\frac{1}{{\left(\rho_{NIR\;}\;-\;0.06\right)}^{2}\;+\;{\left(\rho_{RED\;}\;-\;0.1\right)}^{2}}$	[17]
Sentinel-2 Burn Area Index	BAIS2	$\left(1-\sqrt{\frac{\rho_{Re2\;}\times \rho_{Re3\;}\times \rho_{NIRn\;}}{\rho_{RED\;}}}\right)\;\times \;\left(\frac{\rho_{SWIR2\;}-\rho_{NIRn\;}}{\sqrt{\rho_{SWIR2\;}+\rho_{NIRn\;}}}+1\right)$	[4]
Normalized Burn Ratio	NBR	$\frac{\rho_{NIR\;}-\rho_{SWIR2\;}}{\rho_{NIR\;}+\rho_{SWIR2\;}}$	[10]
Normalized Burn Ratio 2	NBR2	$\frac{\rho_{SWIR1\;}-\rho_{SWIR2\;}}{\rho_{SWIR1\;}+\rho_{SWIR2\;}}$	[15]
Mid-Infrared Burn Index	MIRBI	$10\;\times \rho_{SWIR2\;}-9.8\;\times \rho_{SWIR1}+2$	[26]
Sentinel-2 Tasseled Cap Transformation: Brightness Index	TBI	0.3510 × $\rho_{BLUE}$ + 0.3813 ×$\rho_{GREEN}$ + 0.3437 ×$\rho_{RED}$ + 0.7196 × $\rho_{NIR}$+ 0.2396 ×$\rho_{SWIR1}$ + 0.1949 ×$\rho_{SWIR2}$	[23]
Sentinel-2 Tasseled Cap Transformation: Greenness Index	TGI	-0.3599 × $\rho_{BLUE}$ - 0.3533 ×$\rho_{GREEN}$ - 0.4734 ×$\rho_{RED}$ + 0.6633 × $\rho_{NIR}$ + 0.0087 ×$\rho_{SWIR1}$ - 0.2856 ×$\rho_{SWIR2}$	[23]
Sentinel-2 Tasseled Cap Transformation: Wetness Index	TWI	0.2578 × $\rho_{BLUE}$ + 0.2305 ×$\rho_{GREEN}$ + 0.0883 ×$\rho_{RED}$ + 0.1071 × $\rho_{NIR}$- 0.7611 ×$\rho_{SWIR1}$ - 0.5308 ×$\rho_{SWIR2}$	[23]

### Class separability assessment

Class separability [22] is a reliable measure to test the ability of a spectral index to separate burned from unburned pixels. We calculated the parametric separability index using the M-statistic [9] as follows:

(1) $$M=\frac{\left|\mu_{B}-\mu_{UB}\right|}{\sigma_{B}+\sigma_{UB}}$$

where $\mu_{B}$and $\mu_{UB}\;$represent the mean value of burned and unburned classes and $\sigma_{B}$ and $\sigma_{UB}$ represent the corresponding standard deviations. M values lower than one indicate poor class separability, whereas values higher than one indicate good separability. It is common to have a few trees in the middle of a field, which are generally unaffected by burning. Since it was not always possible to avoid within field trees when delineating burned polygons, we applied a mean NDVI threshold to remove pixels with vegetation that interfered with class separability. Since we observed that pixels representing trees had NDVI values greater than the mean NDVI value for each image in the composite, we masked all pixels that had an NDVI value greater than this mean value. Indices with M values greater than one were selected for further analysis. We then analysed the spectral distribution of all four indices (BAI, BAIS2, NBR and TBI), showing M values greater than one over the two classes, Sampled Burned (S_B_) and Sampled Unburned (S_UB_), to design the threshold conditioning used in subsequent analysis. Further, we excluded NBR from further analysis due to its insufficient class separability despite having an M value higher than one.

### Burn threshold conditioning

**Fig. 3:**
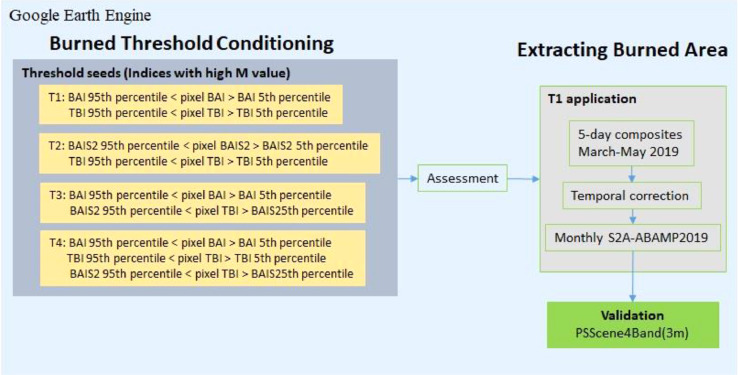
Burned threshold conditioning and extracting burned pixels using T1 threshold condition.

To avoid overlapping pixels and outliers, we set a fixed threshold range for burned and unburned classes to enhance separability. We used the Sampled Burned (S_B_) class limits to detect the burned area from S2A images. Burned scars were separated from the background (unburned areas), assigning the 5th and 95th percentile values of each index as upper and lower thresholds. The threshold values were fixed after considering the spectral distribution (spread of curve, overlapping between classes, outliers etc.) of both sampled classes. The pixel was classified and extracted as burned if it satisfied all of the conditions of the respective threshold combination. Due to the resemblance in detection principle of BAI and BAIS2, we designed three multi-band combinations and examined their individual and combined ability to detect agricultural burned scars. To test the individual performance of BAI and BAIS2, we designed two threshold combinations, T1: BAI and TBI, and T2: BAIS2 and TBI. The third threshold combination, T3: BAI and BAIS2, was used to test the combined performance of all selected indices. We created an additional combination T4 with all three indices to see if TBI can improve T3 performance by bridging the spectral distribution gap between the two indices. The threshold combinations are shown in Fig. 3.

Threshold combinations were applied separately to all 7-day S2A composites of 2019 used for extracting burned and unburned polygons. The results were transformed into binary images (burned: foreground, unburned: background), and the combined outputs were compared to identify the optimal threshold criteria to be used to develop S2A-ABAMP201921 products for 2019 to 2021 post-winter burning season.

### Threshold assessment

All threshold combinations were assessed using the algorithm smileRandomForest (RF) [6]. The same set of burned and unburned sample polygons was used to train a decision forest classifier. RF was used to classify binary images generated after each threshold application. The error of commission (C_E_) was used as a determinant of the performance of the threshold condition. The C_E_ represents the fraction of false positives (i.e. pixels predicted as burned but not actually burned). Since the burned sample pixels used for training the classifier belonged to a binary image, a high C_E_ indicates the low performance of the threshold to extract burned areas from S2A images. The C_E_ is calculated as follows:

(2) $$C_{E}=\frac{\mathrm{FP}}{\left(\mathrm{TP}+\mathrm{FP}\right)}$$

where, FP and FN are false positives and false negatives, and TP are true positives for the given class.

Omission error (O_E_), a measure of false negatives, is calculated as follows:

(3) $$O_{E}\;=\;\frac{\mathrm{FN}}{\left(\mathrm{FN}+\mathrm{TP}\right)}$$

In addition to C_E_ and O_E,_ the F1 score was also calculated, which measures a test's accuracy. The F1 score is the harmonic mean of precision and recall and is more reliable than overall accuracy [12]. The F1 score is calculated as follows:

(4) $$F1=\frac{TP}{TP+0.5\left(FP+FN\right)}$$

where, TP is a true positive, FP is a false positive, and FN is a false negative.

### Burned area map

The T1 threshold combination was applied to all S2A images with cloud cover less than 10 % over the study area to create monthly agriculture burned area maps for the post-winter burning season from 2019 to 2021. An additional cloud filter was applied to the images with shadows over burned scars to avoid false-positive signatures from cloud shadow edges. The traditional way of extracting burned area by two consecutive image subtraction methods was not followed, considering the short-lived burned signature from agriculture fields. Instead, the threshold was applied to all available images, and 5-day composites were created for temporal extraction of burned areas. A continuous three-month temporal correction (see [3] Supplemental Materials: Figure S2) was applied by masking burned area of each 5-day composite (t_n_) using burned areas detected by the previous 5-day composite (t_n-1_) to avoid repeat extraction of long-lasting burned signatures given that the gap between the two composites was not more than 10 days.

### Validation of the ABA product

Validation for the S2A-ABAMP201921 product was performed using PlanetScope images from Planet as independent satellite products. We used the 3 m resampled near-daily four-band (RGB and NIR) corrected imagery from all PlanetScope sensors. Near daily images were provided with radiometric, sensor, and geometric corrections with a tile footprint of approximately 25.0 × 23.0 km^2^ [20]. The validation data at higher spatial and temporal resolution (3 m, ∼daily) than S2A (10 m, 5-days) were used as previous studies have recommended using high spatial and temporal resolution data for validation [2]. A total area of 28,159.76 km^2^ was processed to validate the final S2A-ABAMP201921 10 m product for 2019-2021.

We accessed imagery using the free tier for academic use. Validation was performed using images with the same acquisition dates as our S2A data to avoid temporal mismatch (see [3] Supplemental Materials: Table S1). The virtual sample collection methodology explained in section 3.1 was used to derive approximately 30 reference burned and unburned polygons for each PlanetScope image. We subsetted the S2A imagery to match the spatial extent of each PlanetScope scene, and the T1 threshold condition was applied to each subset. The final S2A-ABAMP201921 subset was converted to a binary image with burned foreground as 1 and unburned background as 0. The RF classifier was built using the PlanetScope reference polygons as training samples and then was used to classify the binary image. We also validated each global burned area product using the same validation dataset for 2019. Error statistics were reported for the study area and period (2019-2021). Availability and temporal distribution of reference data are not uniform across the study period, with 17 reference images for 2019, 2 reference images for 2020 and 4 reference images for 2021. Figure S1 (see [3] Supplemental Materials) shows the validation sites and image distribution over the study area.

## Method validation

### Detection of burned areas

**Fig. 4:**
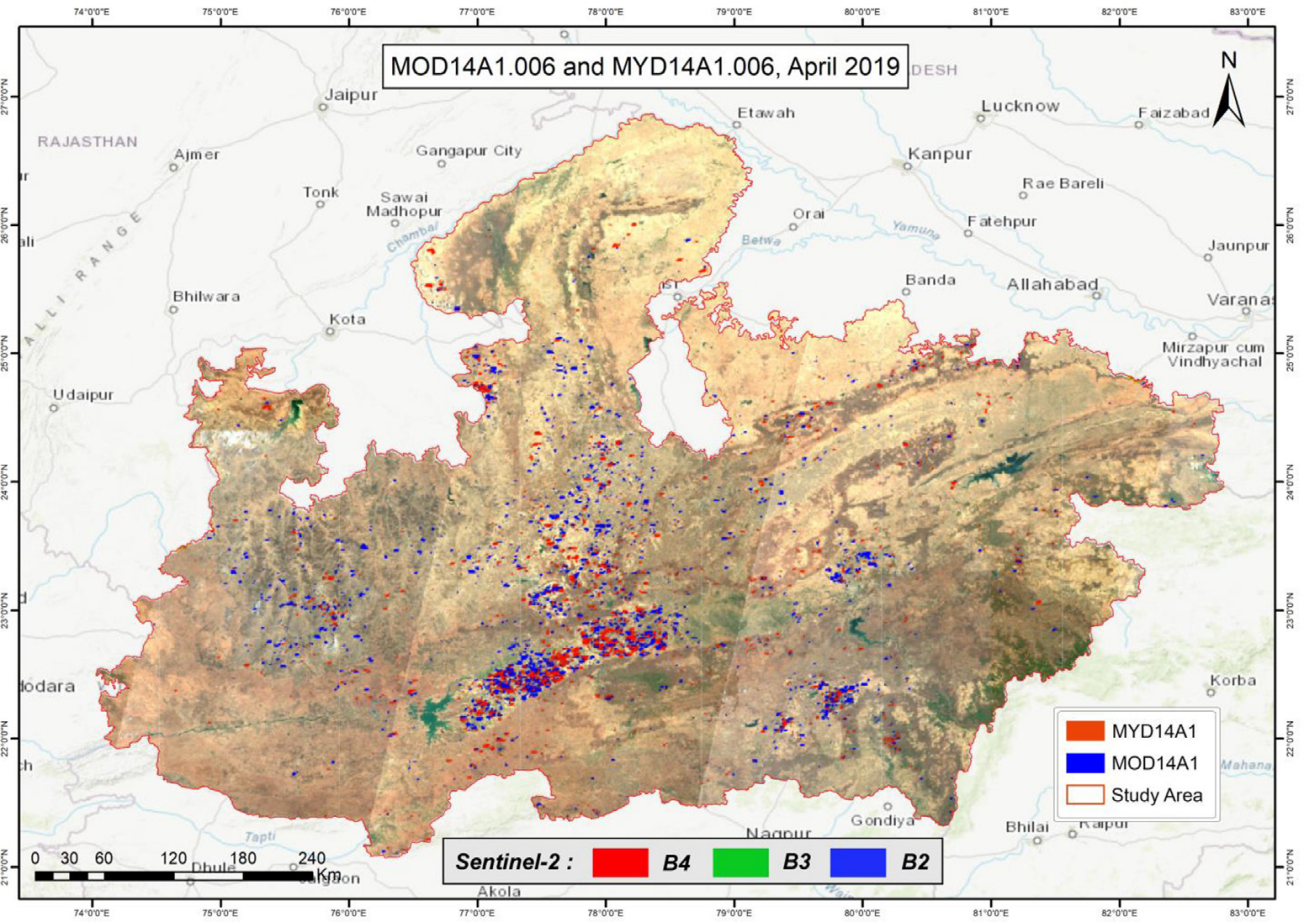
Study area S2A mosaic overlaid with a monthly composite of fire location, April 2019. RGB bands for S2A mosaic at the bottom.

**Fig. 5:**
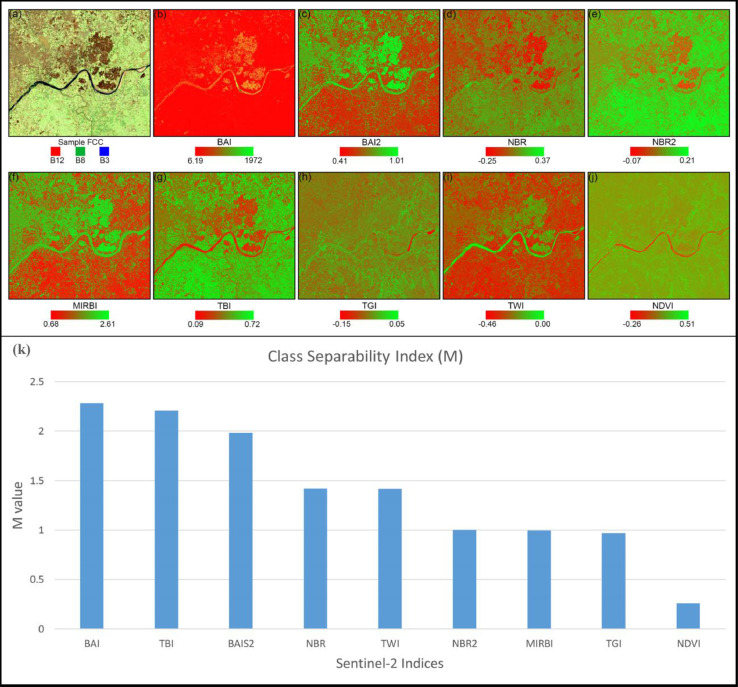
(a) S2A false colour composite (FCC) of reference site. (b) to (j) Performance of different indices in distinguishing burned and unburned areas. The colour bar represents (a) the band combination of FCC and (b) to (j) observed min and max values. (k) Mean M values from all composites used for the sample collection over the study period in descending order of their performance.

**Fig. 6:**
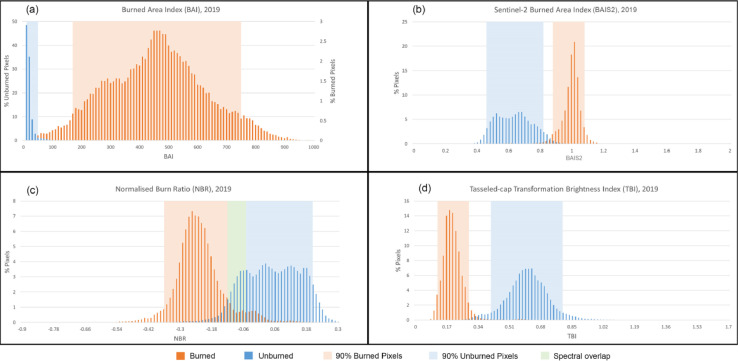
Histogram of spectral signatures from burned (S_B_) and unburned (S_UB_) classes using different fire indices.

Fig. 4 represents the monthly composite of S2A overlaid with monthly composites of MOD14A1.006 and MYD14A1.006 for April 2019, indicating fire activity over the study area. The accuracy of the sampling strategy was assessed by performing the accuracy assessment as described in Section 1.2. The average Cohen's Kappa value is found to be 0.98, (with negligible omission and commission errors of 0.0041 and 0.0039, respectively). Fig. 5 showcases the performance of selected indices with the sample FCC. As expected, none of the indices is able to discriminate between water and burned area. We find that burn indices such as BAI, BAIS2, NBR, NBR2, and MIRBI cannot distinguish between burned areas and wet surfaces (river banks, river islands). However, the TassCap brightness (TBI) and wetness index (TWI) are found to be useful (Fig. 5.) with M values above 1. We select TBI from TassCap indices due to its higher class separability than TWI (M value 2.2 vs 1.4). Fig. 6 shows the spectral behavior of all sample pixels over the sampling period for all four indices. TBI shows a comparatively clear separation of both classes normally distributed with a single prominent peak among the four. Only TBI shows the unimodal distribution with both classes. BAI and BAIS2 show somewhat contrasting behavior with the distribution of S_B_ and S_UB_ signatures. However, BAI shows a more apparent separation than BAIS2; the latter shows a lesser spread with the S_B_ class. Based on our above results, we select BAI, BAIS2 and TBI for threshold conditioning.

**Fig. 7:**
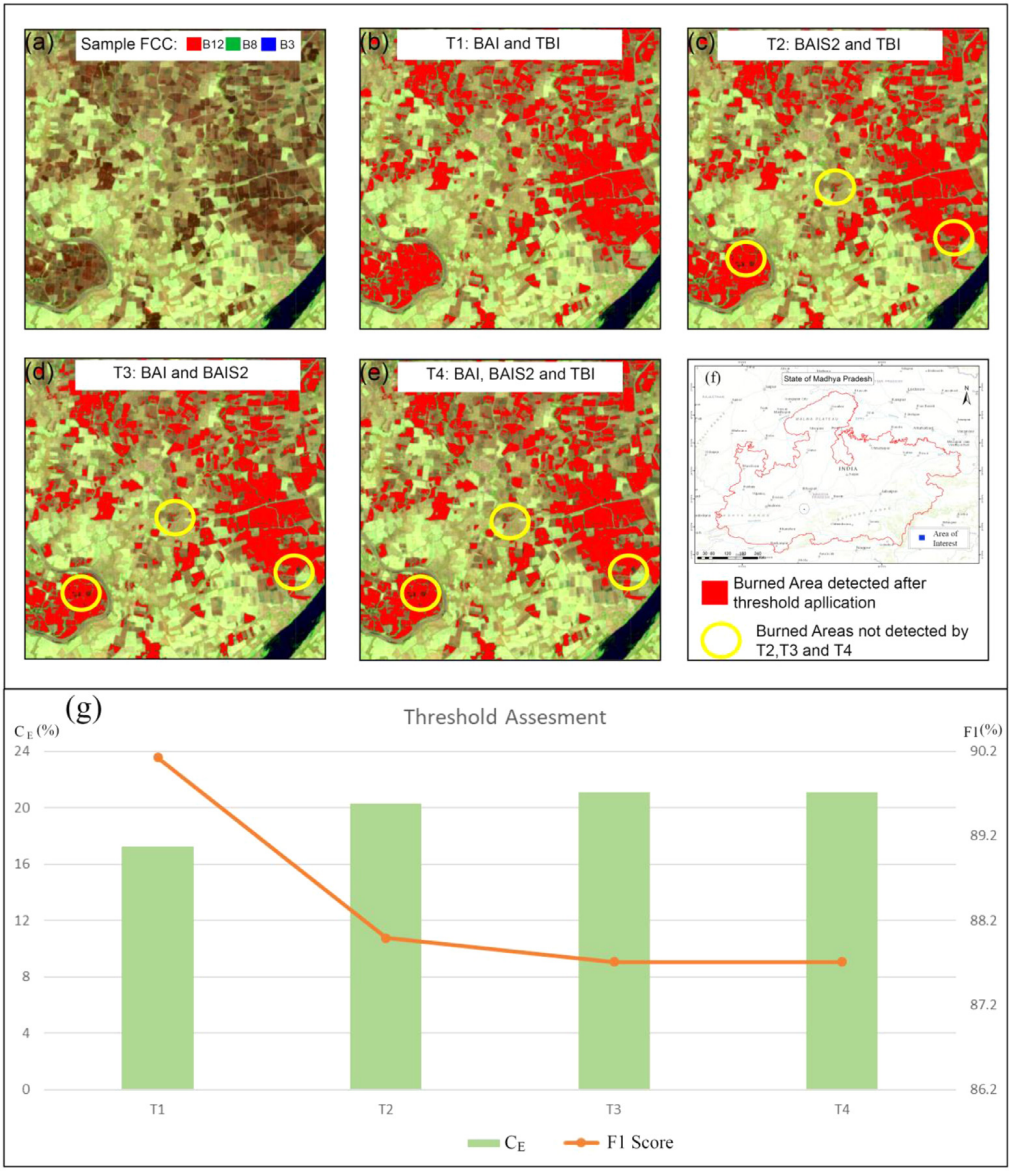
Performance of threshold conditions in burned area detection. (a) S2A false colour composite (FCC) of reference site. (b) to (e) performance of threshold conditions (T1-T4). The yellow circle indicates the false-negative signatures for (T2-T4) against the T1 conditioning. (f) RF-based threshold assessment using burned and unburned training samples. The error of commission (C_E_) is used as a performance measure of threshold condition to provide training pixels for the RF model. F1 score is used to measure model performance for burned areas detected by thresholding. T1 shows a low C_E_ and high F1 score indicating an acceptable threshold condition.

Fig. 7 shows the extracted burned area using different threshold conditions. It can be seen that those threshold combinations other than T1 face limitations due to the narrow spectral distribution for BAIS2. Low C_E_ (∼17%) and high F1 score (∼90%) for T1 indicate better effectiveness in extracting burned area. A continuous three-month temporal correction is applied to the extracted agricultural burned area, which helps to reduce the repetitive extraction and overestimation of burned area (see [3] Supplemental Materials: Figure S2).

### Validation of S2AABAMP201921, MCD64A1 and FireCCI51 using PlanetScope imagery

The accuracy assessment results after comparing S2A-ABAMP201921, with validation data from PlanetScope imagery are shown in Table 3. A high C_E_ of 38.31% is observed in 2020, followed by 2021 (28.88%) and 2019 (10.73%); these C_E_ show an inverse relationship with the number of reference data available. On the other hand, O_E_ show the opposite pattern, with low values in 2021 (0.8%) and 2020 (0.1%). O_E_ for 2019 was very small (0.013%), indicating high accuracy. F1 scores for 2019 (94.24%) and 2021 (82.76%) indicate good agreement, whereas for 2020, F1 scores are moderate (76.07%). Overall, these results indicate a fair agreement between reference data and the BA product, considering the availability of the validation dataset. The validation results are found to be encouraging, with relatively low O_E_ and C_E_ and a high F1 score, even when using limited validation data.

**Table 3:** Estimated validation accuracy of S2A-ABAMP201921 (2019-21).

Product	Commission Error (C_E_), %	Omission Error (O_E_), %	F1 score, %
S2A-ABAMP201921 (2019)	10.73	0.013	94.24
S2A-ABAMP201921 (2020)	38.31	0.1	76.07
S2A-ABAMP201921 (2021)	28.88	0.8	82.76

### Caveats

Our approach has a limitation as the products can be influenced by water pixels and cloud shadows, which look similar to burn scars (see [3] Supplemental Materials: Figure S3), during threshold conditioning. We used multiple methods to mask water pixels, but removing small and seasonal water bodies required careful visual interpretation of imagery. Future work would benefit from identifying additional ways to remove such small and seasonal water bodies, which would reduce false-positive signatures in our ABA product. Considering the impact of cloud shadows, we attempted to minimise the impact of cloud cover and cloud shadows by excluding cloudy images, but doing so reduced the temporal resolution of satellite data. Future work may improve on this by identifying accurate ways to remove cloud and cloud shadow pixels instead of removing entire scenes.

## Discussion

The study adopts a popular strategy of using long-lasting burned signatures to map burned areas [[13], [33], [34]]; however, very few studies have attempted to use complimentary multispectral indices to detect burned areas [[13], [34]]. Each index provides different information; hence considering all indices together improves the class separability. In particular, our approach to identify false-positive signatures is better than approaches using traditional burned indices. Specifically, the combined use of BAI, a charcoal sensitive fire index, and TBI, a proxy index for detecting fire-affected surfaces, minimizes false positives better than methods that rely on only fire index [13,22]. Further, our method is much simpler than other algorithms using multiple decision trees ([22] and [13]), but is more focused on detecting burning in agricultural systems by removing burning that occurs on other landcover types (e.g., forests). The emissions from residue burning over MP for post-winter 2019 were estimated using the agricultural area detected using our method and the results were compared with the GFED4.1s emission inventory [3].

## Declaration of Competing Interests

The authors declare that they have no known competing financial interests or personal relationships that could have appeared to influence the work reported in this paper.

## References

[1] Alonso-Canas I., Chuvieco E. (2015). Global burned area mapping from ENVISAT-MERIS and MODIS active fire data. Remote Sens. Environ..

[2] Boschetti, L., Roy, D.P., & Justice, C.O. (2006). International Global Burned Area Satellite Product Validation Protocol. Retrieved from https://lpvs.gsfc.nasa.gov/PDF/BurnedAreaValidationProtocol.pdf

[3] Deshpande M.V., Pillai D., Jain M. (2022). Detecting and quantifying residue burning in smallholder systems: An integrated approach using Sentinel-2 data. Int. J. Appl. Earth Obs. Geoinf..

[4] Filipponi F. (2018). BAIS2: Burned Area Index for Sentinel-2. Proc. AMIA Annu. Fall Symp..

[5] Google Earth Engine Guide 2021, Scale, Google Earth Engine, accessed 2021-08-01, Scale | Google Earth Engine

[6] Gorelick N., Hancher M., Dixon M., Ilyushchenko S., Thau D., Moore R. (2017). Google Earth Engine: Planetary-scale geospatial analysis for everyone. Remote Sens. Environ..

[7] Huang X., Li M., Li J., Song Y. (2012). A high-resolution emission inventory of crop burning in fields in China based on MODIS Thermal Anomalies/Fire products. Atmos. Environ..

[8] Karra K., Kontgis C., Statman-Weil Z., Mazzariello J.C., Mathis M., Brumby S.P. (2021). 2021 IEEE International Geoscience and Remote Sensing Symposium IGARSS.

[9] Kaufman Y.J., Remer L.A. (1994). Detection of Forests Using Mid-IR Reflectance: An Application for Aerosol Studies. IEEE Trans. Geosci. Remote Sens..

[10] Key C.H., Benson N. (1999). Proceedings Joint Fire Science Conference and Workshop.

[11] Koutsias N., Karteris M. (2000). Burned area mapping using logistic regression modeling of a single post-fire Landsat-5 Thematic Mapper image. Int. J. Remote Sens..

[12] Lipton Z.C., Elkan C., Naryanaswamy B. (2014). Optimal thresholding of classifiers to maximize F1 measure. Lecture Notes in Computer Science (Including Subseries Lecture Notes in Artificial Intelligence and Lecture Notes in Bioinformatics).

[13] Long T., Zhang Z., He G., Jiao W., Tang C., Wu B., Yin R. (2019). 30m resolution global annual burned area mapping based on landsat images and Google Earth Engine. Remote Sens..

[14] Lu B., He Y., Tong A. (2016). Evaluation of spectral indices for estimating burn severity in semiarid grasslands. Int. J. Wildland Fire.

[15] Lutes D.C., Keane R.E., Caratti J.F., Key C.H., Benson N.C., Gangi L.J. (2006). FIREMON: Fire effects monitoring and inventory system. Gener. Tech. Rep. USDA Forest Service, RMRS-GTR-164-CD.

[16] Main-Knorn M., Pflug B., Louis J., Debaecker V., Müller-Wilm U., Gascon F. (2017). Proceedings Volume 10427, Image and Signal Processing for Remote Sensing XXIII; 1042704 (2017).

[17] Martín Isabel M., Chuvieco Salinero E., & Martín Isabel P. (1997). Cartografía de grandes incendios forestales en la Península Ibérica a partir de imágenes NOAA-AVHRR. Serie Geográfica.

[18] NIMA (2000). National Imagery and Mapping Agency(NIMA). Dep. Defense World Geodetic Syst. 1984.

[19] Pinty B., Verstraete M.M. (1992). GEMI: a non-linear index to monitor global vegetation from satellites. Vegetatio.

[20] Planet Team (2018). Planet Application Program Interface: In Space for Life on Earth. San Francisco, CA. https://api.planet.com

[21] Quintano C., Fernández-Manso A., Fernández-Manso O. (2018). Combination of Landsat and Sentinel-2 MSI data for initial assessing of burn severity. Int. J. Appl. Earth Obs. Geoinf..

[22] Roteta E., Bastarrika A., Padilla M., Storm T., Chuvieco E. (2019). Development of a Sentinel-2 burned area algorithm: Generation of a small fire database for sub-Saharan Africa. Remote Sens. Environ..

[23] Shi T., Xu H. (2019). Derivation of Tasseled Cap Transformation Coefficients for Sentinel-2 MSI At-Sensor Reflectance Data. IEEE J. Sel. Top. Appl. Earth Obser. Remote Sens..

[24] Stroppiana D., Boschetti M., Zaffaroni P., Brivio P.A. (2009). Analysis and interpretation of spectral indices for soft multicriteria burned-area mapping in mediterranean regions. IEEE Geosci. Remote Sens. Lett..

[25] Tanase M.A., Belenguer-Plomer M.A., Roteta E., Bastarrika A., Wheeler J., Fernández-Carrillo Á., …, Chuvieco E. (2020). Burned area detection and mapping: Intercomparison of Sentinel-1 and Sentinel-2 based algorithms over tropical Africa. Remote Sensing.

[26] Trigg S., Flasse S. (2001). An evaluation of different bi-spectral spaces for discriminating burned shrub-savannah. Int. J. Remote Sens..

[27] Venkataraman C., Habib G., Kadamba D., Shrivastava M., Leon J.F., Crouzille B., Streets D.G. (2006). Emissions from open biomass burning in India: Integrating the inventory approach with high-resolution Moderate Resolution Imaging Spectroradiometer (MODIS) active-fire and land cover data. Global Biogeochem. Cycles.

[28] Victoria D., de C., da Paz A.R., Coutinho A.C., Kastens J., Brown J.C. (2012). Cropland area estimates using Modis NDVI time series in the state of Mato Grosso, Brazil. Pesquisa Agropecuaria Brasileira.

[29] Wozniak, E., & Aleksandrowicz, S. (2016). An object-based burnt area detection method based on landsat images - a step forward for automatic global high-resolution mapping. (Martin 1998) doi:10.3990/2.459.

[30] Ying L., Shen Z., Yang M., Piao S. (2019). Wildfire detection probability of MODIS fire products under the constraint of environmental factors: A study based on confirmed ground wildfire records. Remote Sens..

[31] Zhang W., Yu M., He Q., Wang T., Lin L., Cao K., Chen J. (2020). The spatial and temporal impact of agricultural crop residual burning on local land surface temperature in three provinces across China from 2015 to 2017. J. Clean. Prod..

[33] Giglio L., Boschetti L., Roy D.P., Humber M.L., Justice C.O. (2018). The collection 6 MODIS burned area mapping algorithm and product. Remote Sens. Environ..

[34] H. Huang, D. Roy, L. Boschetti, H. Zhang, L. Yan, S. Kumar, J. Gomez-Dans, J. Li, Separability analysis of Sentinel-2A Multi-Spectral Instrument (MSI) data for burned area discrimination, Remote Sens., 8 (10) (2016), 873, doi:10.3390/rs8100873.

